# The Strengths of Wisdom Provide Unique Contributions to Improved Leadership, Sustainability, Inequality, Gross National Happiness, and Civic Discourse in the Face of Contemporary World Problems

**DOI:** 10.3390/jintelligence6020022

**Published:** 2018-04-09

**Authors:** Igor Grossmann, Justin P. Brienza

**Affiliations:** 1 Department of Psychology, University of Waterloo, Waterloo, ON N2L 3G1, Canada; 2 Lazaridis School of Business and Economics, Wilfrid Laurier University, Waterloo, ON N2L 3C7, Canada; jbrienza@wlu.ca

**Keywords:** wisdom, reasoning, virtues, well-being, political polarization, culture, social class, egocentrism, leadership

## Abstract

We present evidence for the strengths of the intellectual virtues that philosophers and behavioral scientists characterize as key cognitive elements of wisdom. Wisdom has been of centuries-long interest for philosophical scholarship, but relative to intelligence largely neglected in public discourse on educational science, public policy, and societal well-being. Wise reasoning characteristics include intellectual humility, recognition of uncertainty, consideration of diverse viewpoints, and an attempt to integrate these viewpoints. Emerging scholarship on these features of wisdom suggest that they uniquely contribute to societal well-being, improve leadership, shed light on societal inequality, promote cooperation in Public Goods Games and reduce political polarization and intergroup-hostility. We review empirical evidence about macro-cultural, ecological, situational, and person-level processes facilitating and inhibiting wisdom in daily life. Based on this evidence, we speculate about ways to foster wisdom in education, organizations, and institutions.

## 1. Introduction

As the world is approaching the end of the second decade of the 21st century, human progress in scientific knowledge and medicine has contributed to the lowest levels of disease-based mortality, illiteracy, extreme poverty [1], as well as a shift from patriarchal to emancipative values [2]. For instance, medical advances and success in containing the spread of infectious diseases have been linked to greater gender equality in many countries around the world [3]. At the same time, advances in science and technology have contributed to the increasing complexity of world affairs. Nuclear energy has provided great prosperity to many countries but also provokes worry about the demise of humanity in the case of a nuclear strike. Instant availability and ever-increasing wealth of information through traditional and social media has made us smarter but has also contributed to skepticism of “fake news” and has facilitated partisanship and increasing political polarization in many Western countries [4,5,6,7]. As the world entered the year 2018, the Secretary-General of the United Nations, António Guterres issued a “red alert”. Guterres pointed out a deepening of conflicts, growing inequalities, increasing xenophobia and nationalism, violations of human rights, and global anxieties of nuclear weapons being at the highest since the end of the Cold War [8].

Arguably, the advances in sciences, medicine, and technology reflect shifts in overall levels of human intelligence. At the same time, as the introductory article to this special issue indicates [9], the ever-increasing complexity of social and political affairs suggest that intelligence alone is not sufficient to solve the contemporary, “ill-defined” problems people are facing on the interpersonal and intergroup levels. These problems are ill-defined (or ill-structured) due to numerous unknown parameters preventing an easy formula-based solution to a problem [10]. As a brief glimpse at the history of the last century indicates, technological and medical advances have often been accompanied by massive-scale suffering and misery. Social critiques point out that the same culture that promoted advances in medicine and technology contributed to a breakdown of ecological systems, species extinction, as well as toxic waste and pollution (e.g., [11]). Balancing gains in intelligence and power requires wisdom—a seemingly ancient, yet empirically understudied concept [12]. Infusing wisdom into the public discourse can provide policy-makers with critical tools for addressing ill-defined challenges facing the world today.

### 1.1. A Cautionary Preface

Intelligence and wisdom can have many faces. Even specialist scholars exploring either concept cannot reach perfect agreement on the nuances. Intelligence can mean logic, planning, understanding, learning, reasoning, but also self-awareness, emotional knowledge, creativity, and problem solving (e.g., [13,14]), though much of the mainstream science of intelligence focuses on some common underlying cognitive factor(s) (e.g., [15,16]). Similarly, wisdom can refer to cultural norms and values, intuitions, life experience, autobiographic narratives, emotion regulation, and moral concerns (for reviews, see [12,17,18,19]). In the present article, we use a narrow definition of wisdom, focusing on higher-order cognition. We do this for several reasons. First, by focusing on the reasoning aspects of wisdom affording sound judgment, we aim to unpack key psychological constituents in the Platonian and Aristotelian concepts of wisdom (readily admitting that our narrow attempt will provide only an incomplete portrayal of these philosophical underpinnings [20]). Second, narrowing the focus on cognition allows us to remain in the same realm when comparing psychological processes involved in intelligence and wisdom. Third, cognitive aspects of wisdom appear to be most common to the recent characterizations of wisdom both in psychology and in cross-cultural lay views of wisdom [17] (for an updated review of common characteristics, see [21]). However, we caution the reader that the findings reviewed below do not speak to the notion of wisdom writ large, though arguably some of the processes discussed below may also play an important role for such wisdom-related concepts as effective emotion regulation (for instance, [22], for a review, see [23] and morality, e.g., [24]).

In what follows, we start by drawing a distinction between mainstream views of cognitive processes characterizing intelligence and wisdom in reasoning (wise reasoning from here on). We proceed by highlighting possible societal benefits of wise reasoning for public policy, focusing on five domains: Gross National Happiness, leadership, sustainability, inequality, and civic discourse. At the end, we build on recent evidence about ways to facilitate wise reasoning to showcase how societies can educate for wise reasoning.

### 1.2. Wisdom Complements Intelligence: A Case for Wise Reasoning

At least since Aristotle, philosophers have speculated that people require certain forms of reflection or reasoning when navigating the complex dilemmas and trade-offs they encounter in social life (for a cross-cultural perspective, see [21], for a selective review, see [25]). One may wonder, however, whether philosophic characterizations of such cognitive processes map on to the mainstream definition of intelligence favored in the behavioral and education sciences. For philosophers like Aristotle, superior reasoning is characterized by its wisdom. What does such wise reasoning entail and how is it distinct from the mainstream definitions of intelligence? Like intelligence, wisdom requires at least a basic level of general knowledge and the application of logic. At the same time, philosophers and some behavioral scientists are quick to point out that neither general knowledge nor logic should be confused with wisdom (e.g., [26,27,28,29,30,31,32,33]). Behavioral scientists have proposed that wisdom uniquely involves context-sensitive processing of knowledge, enabling understanding and navigating the complexities of one’s social world [28,30,34]. In ancient Greece, this feature of wisdom has been described by Aristotle as *phronesis* and in the modern scholarship, it is often characterized as a pragmatic capacity to balance and integrate diverse viewpoints in a way that enables one to work through the challenges of social life [35].

When empirical scientists started to become interested in wisdom, scholars were quick to distinguish wise from intelligent (or analytical) reasoning. Scholars like Clayton suggested that mainstream definitions of intelligence focus on abstract symbolic rules and procedures such as propositional logic [30,36]. Abstract logic and other domain-general abilities are advantageous when solving well-structured problems in which all parameters in the evaluative space are known [37]. Thus, in the well-defined situations surrounding financial or technical decisions, features of intelligence such as superior knowledge (e.g., financial literacy, specialized knowledge of physics and engineering) and logic can promote an optimal decision. However, if decisions concern questions of social rather than purely financial or technical nature, these domain-general abilities may be insufficient for a sound decision.

Social issues typically involve other people who may have opinions and interests differing from one’s own—they are ill-structured. Ill-structured problems can concern value trade-offs, unclear means or end-goals, or other situations with incomplete information for a decision [38]. Here, features of abstract reasoning such as symbolic rules and propositional logic can be of little help. Instead of applying a general rule, one may rather benefit from ways to enhance one’s sensitivity to and integration of contextual contingencies. Under such circumstances, abstract cognition may be augmented by metacognitive strategies affording open, nuanced, and dynamic processing of information [17,25,30,36,39,40,41]: Epistemic humility, recognition of varied contexts of life and how they change over time, and open-mindedness toward the possibility of multiple outcomes of a situation and the different viewpoints other people bring to the table (see Table 1).

To illustrate this point, consider the following letter sent to an advice columnist Abigail Van Buren:

My husband is very political, and around election time he becomes engrossed in news shows. He has a habit of showing his favorite political news clips to friends when they visit. I am uncomfortable with this, as I feel our friends are too polite to decline, and they allow my husband to preach politics to them out of courtesy to the host. They are like-minded, politically speaking, and the few who aren’t are not going to be swayed by comedy news shows. I excuse myself from the room when he begins his sermons. I have asked him to stop doing this when friends visit, but he refuses. How can I persuade him to just have “friends time” with no politics?

(adopted from [41])

A wise reasoning approach to this issue may start by realizing that one may not know enough about the husband’s motives or the political issues he aims to promote. One may also consider how such behavior may be temporary, and how the husband acted differently in the past and may again act differently in the future. Finally, one may focus on the perspectives of friends involved in the situation and search for a way to balance both the husband’s and their friends’ interests. As becomes evident, a wise reasoning approach does not necessarily advocate for a single solution. Instead, it facilitates attention to the bigger picture surrounding the situation and the balance of different perspectives and interests.

An intelligent approach to the same problem could take a similar path. Yet, it is equally plausible too that a *self-focused* intelligent person would start searching for the best pieces of evidence in support of one’s request, possibly enumerating the times the husband has demonstrated the disturbing pattern of behavior. Such an approach can result in a fallacy known as a confirmation bias [42], making it antithetical to a wise judgment [43]. Moreover, such an approach can likely backfire, threatening the husband, and possibly motivating him to focus only on friends who endorse his viewpoints or even to start keeping track of all the unpleasant experiences he has with his partner. Instead of bringing the spouses closer and helping figure out a solution that would work for them and their friends, an intelligent approach may, in fact, ruin the respective relationships.

It appears that domain-general cognition does *not necessarily* translate into the context-sensitive processing of information characterized in the wise reasoning approach above. We summarize the common features of the latter approach in Table 1, providing a few examples for a possible manifestation of each feature. Central to these features is their fostering of greater sensitivity to contextual (interpersonal and intertemporal) contingencies, providing greater insight into the complex nature of the uncertain situation at hand. We should note that this conceptualization of wise reasoning aligns with the neo-Piagetian theorizing on features of mature thought in developmental psychology [39,44] and builds on the conceptualization of wisdom-related knowledge advanced by Baltes and colleagues [28,45], avoiding conflation of declarative knowledge with meta-cognitive strategies utilized when working through ill-structured situations/dilemma. It also shared cognitive features with other models of wisdom in the psychological literature [26,46,47] (for a more nuanced comparison of different models of wisdom in behavioral sciences, see [48]).

Using a range of methods to measure these features of reasoning [25,30,41,48,49], the empirical studies provided support the idea that these features of reasoning explain a unique portion of variance on measures of cognitive and personality-related individual differences, showing weak positive relations between wise reasoning and standard measures of intelligence and related physiological processes [43,50,51,52], as well as established individual difference constructs such as the Big Five personality traits (e.g., “openness to new experiences” or “agreeableness”) [49].

## 2. Policy-Making

Wise reasoning can provide unique societal benefits when facing challenges in intergroup relations, at work, and those faced by members of a society in their personal lives. Below, we point to recent evidence suggesting that reasoning aspects of wisdom may be particularly relevant for coping with social challenges of relevance for public policy. We would like to touch on five domains in which we see wisdom-related insights of particular relevance: Gross National Happiness; leadership, sustainability, inequality, and civic discourse.

### 2.1. Gross National Happiness

The economic wealth of a nation does not necessarily correspond to the levels of well-being of people living in a given country. This led several countries such as Bhutan to start exploring ways to facilitate the Gross National Happiness—i.e., the well-being of its citizens [53]. Bhutan is not alone. In 2011, the UN passed a resolution “Happiness: towards a holistic approach to development,” aiming to encourage political leaders to find ways to promote the well-being of their constituents. Worldwide, surveys such as the OECD Better Life Index or the Social Progress Index by the non-profit Social Process Initiative highlight the rising awareness of societal well-being.

Research from the last two decades has begun to identify a range of unique benefits of wise reasoning for improving well-being. One should note that large-scale studies failed to observe a positive relationship between scores on mainstream intelligence tests and well-being (e.g., [54,55,56]), suggesting that rising levels of intelligence in many Western nations do not need to correspond to societal shifts in well-being. In contrast, newer empirical scholarship has started to indicate that having a *wiser* outlook on life can yield benefits to well-being. Higher scores on the wisdom-related characteristics reviewed in Table 1 have been positively linked to reports of greater interpersonal well-being [51], superior emotion regulation [57], and lower intensity of negative emotions [51,58]. Until recently, cross-sectional studies could not yield a conclusive picture concerning the relationship between wise reasoning and positive emotions or life satisfaction [51,57,59,60]. However, new national longitudinal data suggests that among U.S. Americans a wise outlook on life (i.e., being intellectually humble, recognizing change in the world, and considering different perspectives) predicts an increase in positive emotions and life satisfaction over the course of 20 years [61]. Overall, these observations support the philosophical model of wisdom as a set of features that promote a “good” life [17,62,63]. These findings suggest that Gross National Happiness can be promoted by fostering and educating for wise reasoning in a society.

### 2.2. Leadership

We argue that wise reasoning may provide an edge in managing contemporary leadership challenges. Leadership not only requires decision making about regulations and policies. Leaders also serve as models and guides by which business and society can change for the better, thereby impacting people’s values, attitudes, and behavior. Throughout history, leaders who demonstrated epistemic humility and an ability to face up to complexity and change, inspired societal cooperation, and showed concern for the greater good have been marked as most influential, admired, and wise (e.g., Gandhi; Martin Luther King, Jr.) [64]. Contemporary leadership requires wisdom to tackle the challenges of life in the 21st century: the increasing rate of change and uncertainty in business, politics and civic affairs, the need to motivate cooperation among and between increasingly diverse stakeholders, and growing concern for bigger-picture, ethical and socially responsible decision making.

**Wise leadership**. Wisdom-related qualities play a role in overcoming leadership challenges and can contribute to leaders’ outstanding success. As an example, consider that Anne Mulcahy is credited with keeping the Xerox Corporation afloat by successfully navigating the financial and ethical challenges the company faced in the early 2000s. Taking over the CEO role, Mulcahy was advised to take the easy route and declare bankruptcy. Taking a bigger-picture perspective, Mulcahy recognized that such a decision could have ruined the company and any long-term prospects for a viable future. She displayed intellectual humility by personally meeting with stakeholders, allowing them to voice their concerns, heeding advice, taking personal responsibility and apologizing for the company’s past mistakes. She set a firm commitment to ethics, human rights, and sustainable business practice, including righting past wrongs (e.g., in accounting and social irresponsibility). “By doing the right thing for our stakeholders (i.e., more than just stockholders) and the global community,” she said, “we’re also doing what’s right for our business” [65]. Mulcahy has been widely recognized and praised for her actions (e.g., CEO of the year award, 2008), yet humbly defers credit to her colleagues and subordinates, having said that her success “represents the impressive accomplishments of Xerox people around the world.” It appears that Anne Mulcahy’s intellectual humility in the face of complex challenges, accommodation of different perspectives, needs, and values, all played a significant role during the critical moment in allowing her to harness positive outcomes for a company in trouble and herself. The company remained stable for a few years after Mulcahy’s retirement in 2009, fighting an up-fill battle in the post-print digital age.

**Foolish leadership**. One can juxtapose such examples of wisdom in leadership (also see the Fortune’s 2017 “World’s Greatest Leaders” column) [66] with examples of massive failures resulting from leaders’ neglect of wisdom-related qualities and an excessive focus on self-promotion. Notably, many of the examples in Fortune’s 2016 “Most Disappointing Leaders” [67] find their place in the list explained by factors described in the introduction to this special issue as foundations of foolishness—i.e., the opposite of wisdom [9]. These leaders appear to hold high levels of intelligence, yet fail in their jobs, succumbing to numerous fallacies. We discuss some examples below.

Martin Winterkorn, the former chairman of the board of directors of Volkswagen, seems to have fallen prey to the *omnipotence fallacy* (i.e., a belief that one is invulnerable and can do whatever they want), and the ethical disengagement fallacy (i.e., a belief that ethics are essential for others but not the self) in his handling of the company’s diesel fraud case. As Fortune notes, despite a reputation for being a micromanager, Winterkorn denied any wrongdoing or knowledge of wrongdoing. Other examples provided by Fortune similarly fell prey to at least one fallacy. For instance, Michigan Governor Rick Snyder was held responsible for sacrificing public health and safety for economic face-saving and then shifting blame, thereby exhibiting the ethical disengagement fallacy. It appears that the neglect of wisdom in favor of myopic decision making does not support long-term success for the greater community, nor oneself.

**Variability in leadership**. Even the wisest leaders cannot be wise at all times and in all matters. Indeed, one of the most famous examples, King Solomon, was known for both his wisdom and his foolishness in personal life [68]. This asymmetry is evident in many leaders to whom we typically attribute wisdom (e.g., Gandhi, Martin Luther King, Jr., Mother Theresa).

The observation of variability in wisdom may sound paradoxical: After all, is not “true wisdom” stable? Indeed, in many cultures virtue-based qualities like wisdom are linked to the concept of a morally good “true self” [69,70]—a “robust, invariant tendency to believe that inside every individual there is a “true self” calling them to behave in morally virtuous ways” [71]. Notably, this belief is rooted in psychological essentialism, which is a fundamental cognitive bias assuming that “all entities have deep, unobservable, inherent properties that comprise their true nature” [71], and which may not at all reflect the empirical reality of a virtuous characteristic.

**Figure 1:**
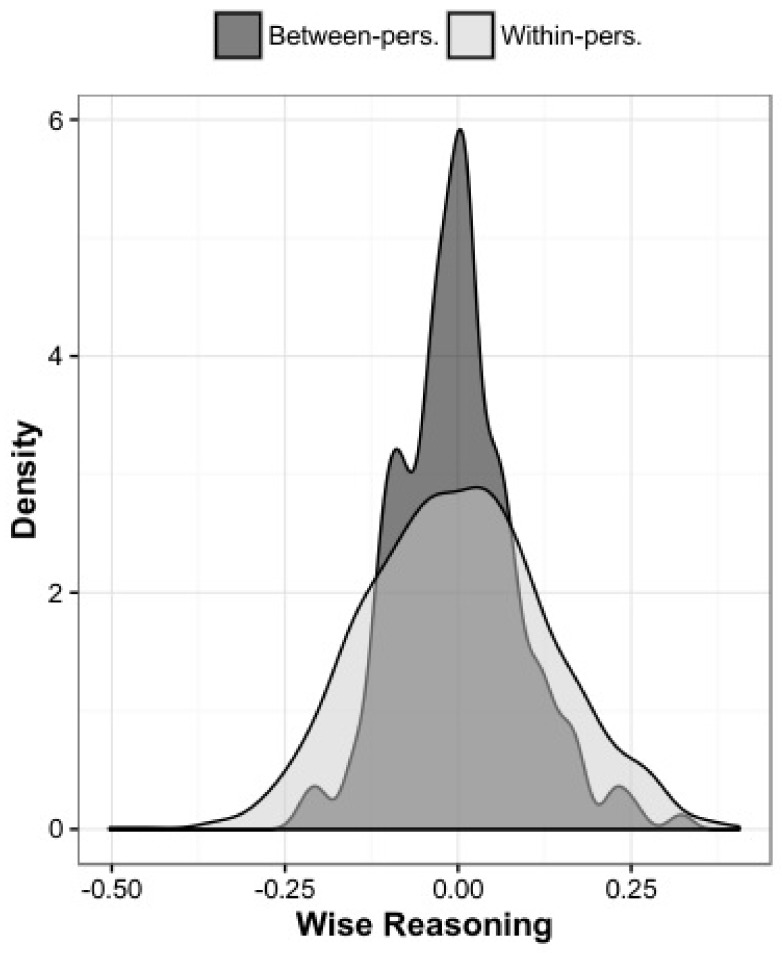
Density distribution of wise reasoning (intellectual humility, consideration of uncertainty/change, perspective-taking) in everyday life, based on reflections about the most challenging issues people encountered across nine days. Within-pers. = Variability of person’s scores from their mean. Between-pers. = Between-person variability in person’s average responses across nine days. Adopted from [58].

Indeed, in everyday life, people’s ability to express wisdom-related epistemic virtues, such as intellectual humility, open-mindedness or the ability to consider a wide range of perspectives on a challenging issue, varies dramatically [57]. As Figure 1 indicates, the variability within a person across several days is at least as large if not larger than the variability between people in their average tendency to express wisdom-related characteristics. This is not to say that there are no trait-level components of wise judgment [49]. Rather, based on the density distribution perspective of individual differences [72], traits may be represented through the unique density distribution profiles of individuals, including unique responses to various situational contingencies [73]. Thus, when discussing wise leadership, is not our intention to discount leaders who are otherwise remarkable and characterize them as fools after singular signs of folly, nor is it our intention to suggest that wise leaders have no faults. Rather, we argue for a pragmatic and evidence-based evaluation of leadership qualities, drawing a connection between the use of wisdom-related attributes and successful leadership (even as judged by sources who are not wisdom scholars), and the relationship between neglect of such attributes and large-scale failure. Moreover, we suggest that awareness of the variability in wisdom-related characteristics and other virtue-based attributes [72] may promote a more contextualized picture of wisdom exemplars in business, politics, and civic discourse.

### 2.3. Sustainability

The current attention to climate change and issues of resource scarcity raise new and urgent questions about how such decisions contribute to integration of short-term and long-term sustainability—i.e., protection of natural resources, while simultaneously improving services and well-being of the most people [74]. The Intergovernmental Panel on Climate Change outlined such concerns in their most recent report, which warned that “delaying global mitigation actions may reduce options for climate-resilient pathways and adaptation in the future” [75].

Questions of sustainability are intertwined with complex social, economic, political, and ecological systems, meaning that they will require more attention to uncertainty, flux, and the bigger picture in which these dilemmas are set [74]. Indeed, as identified at the 2005 World Summit on Social Development, sustainability goals can refer to the balance of economic, environmental, and social goals [76]. Sustainability researchers like Gibson [74] have suggested that a sustainable world view is about intertwined means and ends, embedded in a world of complexity and surprise that requires recognition of links and interdependencies. In Gibson’s view, solutions to sustainability dilemmas depend on context. To craft an adaptive style of sustainability capable of addressing modern environmental issues, one may, therefore, benefit from a capacity for wise reasoning, which directly targets the topics of uncertainties, context, complexities, and multiple perspectives in a proactive manner [30], and can help to identify the balance between such diverse interpersonal, interpersonal, and extrapersonal interests [12,49].

### 2.4. Inequality, Wisdom and Social Class

The driving force behind the global shifts toward greater individualism in industrialized and post-industrial countries appears to involve a rise in the economic prosperity of the country [77,78] (for a review, see [79]). The more affluent a society becomes on average, the higher the shift in the mainstream culture of this society toward greater individualism. Of course, increasing affluence does not equally affect all strata of the population, such that in countries like the U.S., economic inequality is on the rise despite substantial economic growth over the course of the last century [80,81]. Such growing inequality has consequences both for the individual and the society at large, as revealed by many studies concerning the relationship of wisdom and social class.

Research conducted in the U.S., Europe, and East Asia has demonstrated that people with higher socioeconomic status (SES) focus more on the self vs. others [82,83,84,85,86,87], and attend less to contextual features in their social environment [22,88,89].

Drawing on these observations, recent work starts to indicate substantial class differences in the propensity of applying wisdom in reasoning about interpersonal conflicts. Whereas prior research indicated that higher socioeconomic status typically promotes better performance on standardized intelligence tasks (e.g., [90,91]), this newer work starts to suggest a reverse pattern for wise reasoning.

Brienza and Grossmann [92] hypothesized that people with lower (rather than higher) SES would express wiser reasoning about interpersonal conflict situations as it would provide them with an ecological adaptation to secure survival and success in a resource-poor environment. To test their hypothesis, the researchers conducted two studies, involving (i) personality-style assessment of wisdom-related characteristics with a survey on participants’ reflections on recent interpersonal transgressions, and (ii) performance-based assessment of stream-of-thought reflections on standardized interpersonal and intergroup dilemmas [93]. Across both studies, higher SES (Study 1: composite of level of education and income; Study 2: level of education) was associated with significantly lower wise reasoning scores, even when controlling for gender and age, social desirability, emotional intelligence, agreeableness, and abstract cognitive abilities (e.g., executive functioning and crystallized IQ). Moreover, the effect of social class on wise reasoning was at least in part accounted for by a greater sense of social attunement expressed by participants with lower SES.

The observation of social class differences in wise reasoning about interpersonal matters is noteworthy, as it suggests some drawbacks of the contemporary cultural trends in the Western world. As mainstream culture continues shifting toward greater self-focus and individualism, emphasizing uniqueness, individual achievement, and self-serving rationality [94] (but see [95]), it may inadvertently erode wisdom despite the growing complexity of our social world. More specifically, it suggests that people who are more likely to wield the executive power of leadership are especially in danger of making foolish decisions when faced with complex, ill-structured situations. To combat these trends, it appears prudent to (a) allocate more resources to promoting greater inclusiveness of individuals from a broader range of social strata in leadership positions, education, and public policy; and (b) shift societal discourse on social inequality from a “deficiency model,” representing lower social class individuals solely as a “deficient” and “vulnerable” group [96,97,98] to an inclusive model recognizing the unique strengths and vulnerabilities of *each* social strata [99]. Failure to promote inclusivity in such areas as leadership, education, and public policy, and to utilize the strengths and address the vulnerabilities of each social strata, may contribute to further social division, inequality, and societal conflict, and will limit our ability to develop insightful solutions to complex contemporary societal problems.

### 2.5. Civic Discourse

We believe that one of the threats preventing inclusivity on the societal level concerns shifts in political polarization, tribalism, and apathy observed in the civic discourse in North America and Western Europe over the course of the last several decades [4,6,100]. Here we discuss possible ways wise reasoning could be useful for combating these trends.

Political and other group-related polarization has been heightening globally, inflaming intergroup hostilities. Polarization causes clashes and conflicts and threatens societies from making balanced decisions that benefit the greater good (as opposed to single groups). Political and ideological polarization threaten integrative solutions to issues of utmost importance, including health care, inclusivity and effective diversity management, human rights and infrastructure improvements for lower class citizens, immigration and refuge for victims of war, and the list goes on. We suggest that wise reasoning may broaden one’s perspective beyond a limited tribal scope by promoting bipartisanship and attenuating within-group polarization toward solutions that result in shared, collective benefits.

Some initial evidence has shown that wise reasoning may serve such a purpose. As compared to intelligence, wisdom also appears to be uniquely associated with prosocial and eudaimonic tendencies (e.g., cooperative intentions and behavior, growth orientation; [49,101,102,103]), a willingness to forgive friends and family members one has a dispute with [57], as well as more prosocial behavior in economic transactions [104]. Moreover, and particularly pertinent to intergroup issues, wisdom is associated with reduced political bias [105], reduced intergroup attitude polarization across several heightened intergroup conflicts [106] (unpublished manuscript), and the willingness to consider diverse viewpoints during political elections in the US [107], with such aspects of wise reasoning as an appreciation of diverse viewpoints facilitating accuracy in the forecasting of geopolitical events [108].

In several studies, researchers have also found that wise reasoning relates to reduced intergroup bias [106]. The tests were conducted at times of heightened political, ideological, and other group conflicts, each time finding that wise reasoning related to lower or absence of outgroup hostility, improved positivity toward outgroups and moderated “ingroup love.” One study was conducted in the context of the 2015 Baltimore, US, protests, sparked by police violence against Black Americans. In this study, low wise reasoning about the events was linked to extremely unfavorable attitudes toward police among people who identified strongly with the protesters, and unfavorable attitudes toward protesters among people who identified strongly with the police. Conversely, high wise reasoning about the events was linked to less polarized and more balanced intergroup attitudes. This attenuation of polarization did not result in more apathy toward the events or peoples involved: across the different groups, wise reasoning consistently related to greater endorsement that society should use these events as motivation to pursue progress and change rather than the status-quo. Further, reduced polarization via wise reasoning was found to relate to increased acceptance, willingness to associate with, and support for public policy to benefit the (minority) outgroup. These initial findings were replicated across different cultures, ethnicities, and conflicts, and controlling for a host of different demographics (e.g., SES, age, gender) and individual differences in variables known to play a role in intergroup bias (e.g., lay theories of malleability and change in ethnicity).

It is possible that nudging wise reasoning at a societal level could undermine some of the foundations of group polarization. As the classic studies on intergroup conflict indicate, intergroup conflict tends to emerge when members of both groups view resources as a limited zero-sum (for a review, see [109]), whereby resources gained by one party are viewed as losses by another party [110]. By inducing wise reasoning, it is possible that one can shift a view of resources from a zero-sum perspective to a more interdependent, non-zero-sum perspective, as we have recently shown in a related domain of cooperation in a Public Goods Game [104]. It is possible that fostering wise reasoning can help to reduce the tendency to view outgroups as driven by more hate and polarization than ingroups (e.g., [111]), which could result in less reactive intergroup attitudes and behavior (e.g., self-protective aggression). It may also help people to avoid limiting their own perspectives through self-created echo-chambers (e.g., [4]) and increase their open-mindedness to select a broad network of associates with different, more diverse viewpoints.

This emerging work starts to suggest that wise reasoning may play a role for moderating the rampant polarization visible in various parts of the world by broadening people’s purview about who or what is deserving of the care and compassion that groups tend only to give to their immediate ingroup or tribe. We suggest that modeling, nudging, as well as promoting wise reasoning (as compared to self-serving rationality [95]) may allow us to overcome polarization in favor of amicable solutions that benefit society at large, and potentially most significantly to the less fortunate.

## 3. Paths to Wisdom

The unique strengths of wisdom highlighted above suggest potential applications to contemporary individual and societal challenges. They also suggest directions for future research in such domains as leadership, education, and sustainability, and promoting inclusive social-organizational policies and programs meant to maximize the benefits (e.g., progress and innovation) and reduce the pitfalls (e.g., bias and conflict) of an increasingly diverse society. We discuss some of these applications and future directions below.

### 3.1. Education

Is it possible to educate for wisdom in handling contemporary business and societal problems? Business schools provide courses in organizational behavior and human resource management that attempt to engage students in critical thinking (e.g., about their own biases) and communicate balanced, ethical decisions that benefit the self and the greater good. However interesting and well-meaning these courses may be, their effectiveness for guiding wise decision making may be occluded by a hard focus on the notion of economic, self-interested rationality [95,112] as a chief basis for sound judgment in the majority of courses students are required to complete (e.g., [94]). Social critiques suggest that such unitary focus on de-contextualized, self-interested reasoning and decision-making is widely spread in the Western societies, including in school curricula [113,114,115], poorly preparing students for facing the uncertainties and complexities of the ill-structured social world. Indeed, empirical work indicates that Western education promotes higher performance on tests of de-contextualized intelligence, but does so at the expense of fostering social responsibility [116].

It may be the case that what is missing in training is the notion of *reasonableness* as discussed by modern philosophers [117,118,119,120], which views just decisions as those that balance economic pursuits with humility and concern for the common good. We suggest that instruction on the benefits of wise reasoning (e.g., for education, management and leadership)—care and attention to, and integration of, different perspectives and needs, intellectual humility and acknowledging uncertainty and change, and a bigger-picture outlook—may provide a necessary toolkit that students can use to balance self-interested and cooperative goals. Based on the recent empirical evidence, below we discuss potential methods for inducing or training wise reasoning.

**Fostering wisdom by reducing egocentrism**. Over the course of the last decade, research has repeatedly shown that one factor can profoundly impact wise reasoning. This factor concerns the degree to which a person focuses on the self. Both in observational and experimental studies, greater self-focus has led to a lower expression of wisdom-related characteristics. For instance, examining diary entries on the most challenging events of a day revealed that people are less likely to reason wisely when they were surrounded by strangers compared to situations involving co-workers, family, or friends as well [57]. Similarly, when presented with interpersonal transgression scenarios concerning infidelity and trust betrayal, people show lower wisdom when transgressions involve them personally as compared to transgressions involving a close friend [121]. In such scenarios, one is particularly in danger of inhibiting one’s wisdom about interpersonal and intergroup issues if approaching the situation from an egocentric, first-person perspective (as compared to a third-person perspective [107,121,122]).

The observation that greater self-focus inhibits one’s ability to approach interpersonal and intergroup matters wisely may be bad news, given repeated observations about the rise of individualism, self-centeredness, and other related tendencies in many parts of the world. In the US alone, analyses of cultural products such as themes in books, baby naming practices, or household make-up patterns indicate that themes emphasizing personal achievement, uniqueness, preference for single child household, and divorce rates have been on the rise for a good part of the 19th and 20th centuries [77,123,124]. Similar patterns can be observed in other parts of the world, as well [78].

### 3.2. Cultivating Wisdom

How does one grow wisdom in a time of cultural shifts to more individualism? Randomized control-trial studies of wise reasoning suggest several promising ways of fostering wise reasoning under such conditions, as demonstrated by experimental shifts in wise reasoning about personal challenges involving politics, career choices, or interpersonal conflicts. In one set of studies, Kross and Grossmann [107] instructed participants who were pre-screened for polarized political attitudes to reflect on a contentious political issue concerning the election of a candidate they do not endorse as the next U.S. President. Researchers experimentally assigned half of the participants to adopt a perspective of a U.S. citizen living in the U.S. when reflecting on this issue (psychologically close group). The other half of participants were instructed to adopt a perspective of an Icelandic citizen living in Iceland (psychologically distant group). This simple shift in perspective resulted in a higher degree of epistemic humility and view of the situation as in flux/change, as well as promoting greater open-mindedness, shown through their willingness to meet and discuss political issues in a bipartisan fashion.

In other similar studies, researchers examined the effects of adopting a first- (i.e., psychologically close) vs. third-person (i.e., psychologically distant) perspective when reflecting on their career development at the peak of the 2008 economic recession [107], or when reflecting on the possibility of an infidelity by their partner or a trust betrayal by a close friend [121]. In each of these studies, adopting a psychologically distant perspective resulted in wiser reasoning as compared to adopting a psychologically close perspective. Though this effect is not large, it appears to be reliable and extends to people’s reflections on conflicts they experience in their lives [122,125]. Insights about ways to facilitate wisdom-related qualities in the face of political, interpersonal, and personal adversity suggest a possibility of fruitful educational and training programs. For instance, Grossmann [40] has outlined several possibilities for integrating insights about the effects of social and psychological distance on wisdom in the context of educational curricula, though the effectiveness of these and other similar ideas [126] have yet to be evaluated empirically.

Insights about the strategies for promoting wise reasoning suggest that structural changes in the environment and framing of behavioral choices in ways that can encourage wise reasoning in decision making are effective. Similar to “nudges” promoting saving and morally-conscious behavior [127,128], it is foreseeable to develop nudges for wise reasoning through an altering of the structure of organizational and political decision-making. For instance, based on the insights about how solitary decisions (compared to decisions made in the presence of people one cares about) may be less likely to foster wise reasoning, organizations may structure their decision-making environments in a way that would promote interdependent (rather than independent) decisions. For example, organizations could assign individual agents into mentor-mentee pairs to create environments encouraging an open debate among various stakeholders. In such situations, mentors may be more likely to approach contentious issues in a perspective-diverse, open-minded fashion [129]. In situations where solitary decisions are unavoidable, one can also attempt to institute a checklist reminder similar to how checklists are employed to reduce error in medical decision-making [130]. Finally, one can develop context-specific reminders of wisdom-boosting strategies [68] (for a general notion of “boosts,” see [128]). We suggest that particularly promising reminders to specific critical events through computerized communication platforms, employing natural language processing and AI algorithms to detect the significance of a communicated message and providing customized, wisdom-fostering reminders to take a step back, consider a wide range of perspectives, and estimate the degree of uncertainty and the consequences of one’s decision vis-à-vis plausible alternatives are prudent strategies.

Although these findings and speculations provide some hopeful suggestions for improving wisdom at the societal level, their effectiveness would surely depend on the “political will,” and other cultural factors such as a generalized normative acceptance of interdependent decision making and behavior. The current trends in the post-industrial and developing countries indicate that the rise of individualistic attitudes and behavior [78] could represent a mounting hurdle to achieving these ends. Thus, whether these insights about the potential benefits of wise reasoning are able to pan out likely depends to some extent on the societal realization that individual decisions cannot be taken out of context. Recent work suggests that such a realization is growing in certain political circles (e.g., sustainability [76]), giving hope that the rise of individualism will be balanced by an increased realization of systemic interdependence and a greater propensity for wise reasoning.

## 4. Conclusions

A societal focus on increasing domain-general cognitive abilities (as measured by mainstream intelligence tests) has brought welcome improvements to the world in many domains such as health and technology. However, such forms of intelligence alone appear insufficient when facing large-scale social problems involving intergroup conflicts, sustainability concerns, inequality, as well as ill-defined challenges people encounter in their lives. In the current paper, we distinguished mainstream definitions of intelligence from wise reasoning, proposing that the latter concept is useful for working through societal problems. We bolstered our proposition by drawing on recent empirical evidence on the role of wisdom-related processes in deliberation and judgment about social issues. Features of wise reasoning, such as intellectual humility, the recognition of uncertainty and change, a consideration of diverse perspectives, and the search for an integration of these perspectives can promote societal well-being, and they can improve leaders’ ability to provide and guide others toward outcomes that benefit both themselves and the greater good. Further, wise reasoning may help to face societal issues concerning sustainability, inequality, and polarization of the civic discourse. Evidence-based insights start to pave ways to promote wise reasoning in education and strategic decision-making. Whether these insights can be implemented depends on the political will and the societal realization that individual actions can rarely be taken out of social context, making knowledge about ways to situate individual actions into a given situation paramount.

## Figures and Tables

**Table 1:** Features, definition, and possible manifestations of wise reasoning in everyday life, represented by frequently co-occurring aspects of cognition.

Feature	Definition	Possible Manifestation
Intellectual humility	Recognition of limits of one’s knowledge	Double-checking whether one’s opinion on the situation might be incorrect. Searching for extraordinary circumstances before forming an opinion
Recognition of uncertainty and change	Recognition that contexts change over time; open-mindedness about direction of change	Searching for different solutions as the situation evolves Considering alternative ways a situation may unfold
Perspective-taking of diverse viewpoints	Open-mindedness toward different viewpoints on an issue	Making effort to take the other persons’ perspective(s) Taking time to get different opinions on the matter before coming to a conclusion
Integration of different viewpoints	Search for a compromise between different interests at stake for the issue	Considering whether a compromise is possible in resolving the situation Searching for a solution that could result in most of the interests being satisfied (acknowledging that this may not always be possible)
